# Efficacy of a novel thermo-reversible wound gel against antibiotic tolerant biofilm

**DOI:** 10.3389/frabi.2026.1773630

**Published:** 2026-03-12

**Authors:** Jeyachchandran Visvalingam, Anna Muzaleva, Miloslav Sailer, Sarvesh Logsetty, Robert B. Huizinga

**Affiliations:** 1 Kane Biotech Inc, Winnipeg, MB, Canada; 2 Departments of Surgery, Psychiatry, and Children’s Health, Rady Faculty of Health Sciences, University of Manitoba, Winnipeg, MB, Canada

**Keywords:** antibiotic tolerance, biofilm, PHMB, polyhexanide, thermo-reversible

## Abstract

Chronic wounds are frequently colonized by biofilm-forming bacteria, and one of the defining characteristics of these infections is the resulting tolerance to antibiotics. A novel thermo-reversible antimicrobial wound gel (revyve^®^ Antimicrobial Wound Gel, TRG), formulated to target biofilms, was evaluated for its ability to inactivate antibiotic-tolerant biofilms using both a colony biofilm model and a porcine skin explant biofilm model. Mature biofilms of *Staphylococcus aureus* and *Pseudomonas aeruginosa* were grown on nitrocellulose membranes or porcine skin explants for 72 hours at 37 °C. Before any treatment, viable numbers of *S. aureus* and *P. aeruginosa* were ≥ 9.7 log CFU in the colony biofilm model, and 8.3 and 6.6 log CFU, respectively, in the porcine skin explant model. Biofilms were then washed and treated with defined concentrations of antibiotics for 24 hours to select for antibiotic-tolerant cells, followed by up to 7 days of TRG treatment. Antibiotic treatment caused a significant (P ≤ 0.05) reduction in viable numbers of both organisms in both models, resulting in survival of ≥ 5 log CFU of tolerant biofilm cells. Subsequent treatment with TRG reduced viable numbers of *S. aureus* to below detection limits, causing a 7.9 log CFU reduction at 24 hours in the colony biofilm model and a 5.5 log CFU reduction at 72 hours in the porcine skin explant model. In the colony biofilm model, viable numbers of *P. aeruginosa* were reduced to below the detection limit, corresponding to a 6.1 log CFU reduction at 24 hours, while in the porcine skin explant model, TRG caused a 3.5 log CFU reduction at 72 hours, with no further changes observed up to 7 days. These results indicate that TRG was effective at inactivating antibiotic-tolerant biofilms and may serve as a valuable tool in combating biofilms in chronic wounds.

## 1 Introduction

Wound healing involves multiple overlapping processes, including hemostasis, inflammation, tissue proliferation, and remodeling. When these processes are disrupted for various reasons, wound healing can be delayed, leading to chronic wounds (Han and Ceilley, 2017). Chronic wounds are generally defined as those that do not progress through the normal healing process within an expected time frame. Although there is no consensus on the exact duration, it is typically considered to be between one and three months (Werdin et al., 2009; Lazarus et al., 1994; Järbrink et al., 2017). A key feature of these wounds is that they remain in a prolonged inflammatory phase (Werdin et al., 2009). Biofilms have been implicated as a key contributor of wound chronicity (Bjarnsholt, 2013; Bjarnsholt et al., 2008) and have been reported to be present in 80% of chronic wounds including diabetic foot ulcers, venous leg ulcer and pressure ulcers and burn wounds (Malone et al., 2017; Shen et al., 2025).

Bacteria can exist as free-floating single cells, defined as planktonic, or as multicellular aggregates embedded in a self-produced matrix, with or without attachment to a surface, defined as biofilms (Liu et al., 2024; Versey et al., 2021). The self-produced matrix, also referred to as the extracellular polymeric substance (EPS), contains water, polysaccharides, extracellular DNA, proteins, lipids, biosurfactants, flagella, and pili. This matrix provides protection against harmful environmental conditions, such as antimicrobials (including antibiotics) and the host immune system (Bjarnsholt, 2013). Protection against antibiotics may involve binding by charged components, enzymatic inactivation, or acting as a permeability barrier. In addition, biofilm-associated bacteria exhibit distinct gene and protein expression profiles compared to their planktonic counterparts, and their metabolic activity can vary depending on their location within the biofilm. Cells at the periphery of the biofilm are generally more metabolically active, whereas deeply imbedded cells are largely slow-growing, metabolically inactive, or dormant. These slow-growing or dormant cells include “persister” cells, which are tolerant to antibiotics (Liu et al., 2024). This characteristic is distinct from genetically mediated antimicrobial resistance and tolerant cells can revert to an actively growing state once favorable conditions are restored like cessation of antibiotic treatment and repopulate wounds within 24h. Biofilm, therefore, is a complex and resilient biological entity, composed of extracellular polymeric substances (EPS), permeability barriers, and persister cells. Its presence in wounds contributes significantly to recalcitrant infections and treatment failures (Goswami et al., 2023; Liu et al., 2024). Thus, treatment strategies that break up permeability barrier and inactivate persister cells are needed for improving healing outcome of chronic wounds.

Despite evidence indicating that biofilms and persister cells play a key role in wound chronicity and treatment failure, many studies evaluating the effectiveness of wound care products have not assessed their activity against persister cells (Lipp et al., 2010; Robertson et al., 2024; Watson et al., 2025). Furthermore, *in vitro* models, such as polystyrene multi-well plates used to assess the efficacy of wound care products, lack the complexity of the wound environment, which may lead to inconsistencies between results obtained in these systems and real-world outcomes (Pranantyo et al., 2024; Kostenko et al., 2010; Brackman and Coenye, 2016; Outomuro Ruiz et al., 2024). Therefore, selecting an appropriate biofilm model system is essential for accurately evaluating the efficacy of wound dressings. The objective of this study is to assess the effect of a novel thermo-reversible wound gel (TRG) on antibiotic-tolerant biofilms formed by the most common wound pathogens, *Staphylococcus aureus* and *Pseudomonas aeruginosa*, using *in vitro* and *ex vivo* biofilm model systems that better reflect the wound environment (Wolcott et al., 2016; Uberoi et al., 2024; Yang et al., 2013; Hammond et al., 2011).

## 2 Materials and methods

### 2.1 Test product

A novel thermo-reversible wound gel (TRG; revyve^®^ Antimicrobial Wound Gel, Kane Biotech Inc, Winnipeg, MB, Canada) containing chelators, poloxamer 407, and polyhexanide was used in this study (Visvalingam et al., 2024; Gawande et al., 2018). The composition of TRG provides strong antimicrobial and antibiofilm properties that can last for up to 7 days, decreases surface protease levels, works across Gram +ve/-ve organisms along with fungi, and has a thermo-reversible characteristic that enables the gel to conform to wound contours upon application (Visvalingam et al., 2026).

### 2.2 Bacterial culture preparation

*S. aureus* (ATCC 6538) and *P. aeruginosa* (ATCC 15422) were obtained from Cedarlane Labs (Burlington, ON, Canada). Trypticase soy broth (TSB; Becton Dickinson; Fisher Scientific, Ottawa, Ontario, Canada) and trypticase soy agar (TSA; Becton Dickinson; Fisher Scientific) were used as liquid and solid media, respectively. All microbial cultures were stored at −80 °C as stock cultures in appropriate broth containing 15% glycerol (Sigma Aldrich, Oakville, ON, Canada). Working cultures were maintained on an appropriate agar medium (above) at 4 °C with monthly transfer for a maximum duration of 3 months. A single colony from a streaked plate was transferred to 5 mL of an appropriate broth medium and incubated overnight (16–18 h) at 37 °C for all organisms. Mid-logarithmic phase cultures were prepared by transferring 50 µL overnight culture into 5 mL TSB and incubating at 37 °C and 150 rpm to absorbance at 600 nm between 0.2 and 0.6. Log phase culture was diluted in TSB to obtain ~10^6^ CFU/mL for minimal inhibitory concentration (MIC) determination and biofilm experiments.

### 2.3 Determination of minimal inhibitory concentration

MIC of gentamicin and oxacillin was determined for *P. aeruginosa* and *S. aureus*, respectively using broth microdilution assay with modifications (EUCAST and ESCMID, 2003; Nascente et al., 2009). As biofilm experiments were performed using TSB and TSA media, TSB was used for determination of MIC. Effective gentamicin (Sigma Aldrich) and oxacillin (Sigma Aldrich) concentration in microwell plates ranged from 1.56 to 200 µg/mL, and from 0.0156 to 2.0 µg/mL, respectively. Testing was conducted in triplicates and repeated at least twice. Positive control (growth), as well as negative media and antibiotic controls were included in each experiment. Plates were incubated at 37 °C in air for 16–20 h. The amount of growth in each well was compared with that in the positive control, and the MIC was recorded as the lowest concentration of the agent that completely inhibited growth.

### 2.4 Effect of TRG on antibiotic tolerant colony biofilm

The colony biofilm assay described previously for assessing wound related biofilms was used with modifications (Hammond et al., 2011; Qu et al., 2020). Overnight cultures of *S. aureus* and *P. aeruginosa* were prepared as described above and diluted 100-fold in the appropriate broth. Sterile nitrocellulose membranes were placed in each well of a 12-well plate containing 2 mL of TSA. A 10-µL aliquot of the diluted culture was inoculated onto each sterile membrane. The plates were incubated at 37 °C for 72h to allow mature biofilm formation (Jo et al., 2024). Two sets of three *S. aureus* and *P. aeruginosa* biofilm-containing membranes were then treated with 3.13 µg/mL (50× MIC) oxacillin and 312.5 µg/mL (50× MIC) gentamicin, respectively, for 24h at 37 °C. The same number of untreated controls was maintained. After antibiotic treatment, each membrane was washed twice in 2 mL phosphate buffered saline (PBS), and three membranes were transferred to new 12-well plates. Then, 1 mL of gel (TRG) was applied to each antibiotic-treated membrane, forming an approximately 2–3 mm thick layer over the biofilm (Jimenez et al., 2017) and incubated 24h at 37 °C. Viable counts of biofilm-associated bacteria were determined before antibiotic treatment, after antibiotic treatment, and after TRG treatment. Viable counts of untreated controls were also determined at the same time. To determine viable numbers, each biofilm membrane was washed twice in 2 mL PBS to remove planktonic and loosely attached cells. Each washed membrane was then placed into a 14 mL tube containing 2 mL of PHMB-neutralizer solution and 5 mm glass beads. The tubes were vortexed at maximum speed for 1 min to detach biofilm-embedded cells. The resulting suspensions were serially diluted and plated onto the appropriate agar medium. Plates were incubated at 37 °C for 24h, and viable counts were obtained. Detection limit of plating method was 10 log CFU. All experiments were performed two independent times, with triplicate technical replicates in each run (total n = 6).

### 2.5 Preparation and sterilization of porcine skin explants

Sheets of porcine skins with a thickness of 2–4 mm were obtained from HyLife Foods, (Neepawa, Manitoba, Canada) a facility licensed by the Canadian Food Inspection Agency and stored at -20 °C until used. Circular explants of 12 mm in diameter were excised from the skin tissue using a biopsy punch (Acu-Punch; Surgo Surgical Supply, Newmarket, Ontario, Canada). Partial thickness wounds of approximately 2 mm in diameter and a 1.5-mm deep were created using a Dremel tool (Mastercraft, Canadian Tire, Welland, Ontario, Canada). Explants were sterilized using the method described by Yang et al. (2013) with modifications. Briefly, 10 explants were rinsed for 10 min using sterile 40 mL of PBS and explants were transferred to a sterile 50 mL centrifuge tube. Explants were washed at room temperature and on a shaker (SteadyShake757L Incubator Shaker, Amerex Instruments, Inc. Concord, CA, USA) operating at 50 rpm for 30–45 min in 40 mL, 0.6% bleach prepared in PBS containing 5 µL/mL Tween 80 (Sigma Aldrich, Oakville, ON, Canada). Then, explants were transferred into a 50 mL centrifuge tube containing 40 mL of 70% denatured ethanol prepared in PBS containing 5 µL/mL Tween 80 and washed as before. Final wash carried out in 40 mL PBS containing 5 µL/mL Tween 80 as before and then stored in 20 mL PBS containing 5 µL/mL Tween 80 at 2-8 °C for up to a week until used for biofilm experiments.

### 2.6 Optimization of biofilm growth media composition

As indicated by Yang et al. (2013) soft-TSA containing 0.5% agar (w/v) (Fisher Scientific) was used for optimization with modifications. For *P. aeruginosa* biofilm optimization, soft-TSA was prepared with gentamicin concentrations of 62.5, 93.25, 156.25, and 312.5 µg/mL. Each well of a 12-well plate was filled with 2 mL of gentamicin-supplemented soft-TSA and allowed to solidify. Six sterilized explants were individually placed in each well containing different gentamicin concentrations. The wound bed of each explant was inoculated with 10 µL of log-phase bacteria and incubated at 37 °C for 72 hours. Visual biofilm growth on the wound bed and agar surface was assessed, and three explants were processed to determine viable bacterial counts. No visible or viable bacterial growth was observed, suggesting that all tested antibiotic concentrations effectively prevented biofilm formation. To further investigate, explants were first incubated for either 4 or 24h on non-antibiotic soft-TSA at 37 °C to allow initial bacterial growth, and then transferred to soft-TSA containing different gentamicin concentrations for an additional 48 hours at 37 °C. These explants were assessed for visible biofilm formation and viable bacterial counts. Only the condition involving 24-hour pre-incubation on non-antibiotic soft-TSA followed by 48-hour incubation on soft-TSA containing 62.5 µg/mL gentamicin supported biofilm growth. Therefore, this condition was adopted for subsequent experiments.

Similarly, direct incubation of inoculated explants on soft-TSA containing 0.625, 1.56, and 3.13 µg/mL oxacillin did not result in any visible or viable bacterial growth. Therefore, explants were first incubated for either 4 or 24h on non-antibiotic soft-TSA at 37 °C to allow initial bacterial growth, and then transferred to soft-TSA containing different oxacillin concentrations for an additional 48 hours at 37 °C. These explants were assessed for biofilm formation and viable bacterial counts. Only the condition involving 24-hour pre-incubation on non-antibiotic soft-TSA followed by 48-hour incubation on soft-TSA containing 0.625 or 1.56 µg/mL oxacillin supported biofilm growth. Consequently, 24-hour pre-incubation on non-antibiotic soft-TSA and followed by 48h incubation on1.56 µg/mL oxacillin containing soft-TSA was selected for further experiments.

### 2.7 Effect of TRG on total biofilm

*P. aeruginosa* and *S. aureus* biofilms were grown on explants as described above. Each well containing an explant was then treated with 2 mL of TRG, forming a 2–3 mm thick layer of gel over the biofilm/explant surface (Jimenez et al., 2017) for up to 7 days at 37 °C. Untreated control wells were also maintained. On days 1, 3, and 7, three TRG-treated explants and three control explants were washed twice with 2 mL of PBS to remove loosely attached cells. Each explant was then transferred to a 15 mL centrifuge tube containing 7 mL of PHMB neutralizer. The tubes were sonicated for 30 seconds and vortexed at maximum speed for 1 min to dislodge and disperse biofilm-embedded cells. The resulting suspensions were serially diluted and plated onto the appropriate agar media. Plates were incubated at 37 °C for 24h after which viable cell counts were determined. Detection limit of plating method was 10 log CFU. All experiments were performed three independent times, with triplicate technical replicates in each run (total n = 9).

### 2.8 Effect on TRG on antibiotic tolerant biofilm

*P. aeruginosa* and *S. aureus* biofilms were grown on explants as described above. The explants containing biofilms were then treated for 24h at 37 °C with 2 mL of 62.5 μg/mL gentamicin, and 1.56 μg/mL of oxacillin, respectively. Each antibiotic-treated explant was washed three times with 7 mL of PBS and transferred to microwell plates containing soft-TSA with the same concentration of antibiotics. Each explant was then treated with 2 mL of TRG and incubated for up to 7 days at 37 °C. Untreated control wells were also maintained. Viable counts of biofilm-associated bacteria were determined using three explants before antibiotic treatment, after antibiotic treatment, and on days 1, 3, and 7 following TRG treatment. Three explants were processed as described above for each time point. All experiments were performed three independent times, with triplicate technical replicates in each run (total n = 9).

### 2.9 Data analysis

Biofilm-based bacterial counts (log CFU) and porcine wound healing data were analyzed using one-way ANOVA (GraphPad Prism 10 Software, Boston, MA, USA) and are expressed as the mean ± standard deviation (SD). *Post hoc* Tukey’s tests were used to assess differences between means, and a *p*-value ≤ 0.05 was considered statistically significant.

## 3 Results

### 3.1 Effect on antibiotic tolerant biofilm using colony biofilm model

**Figure 1:**
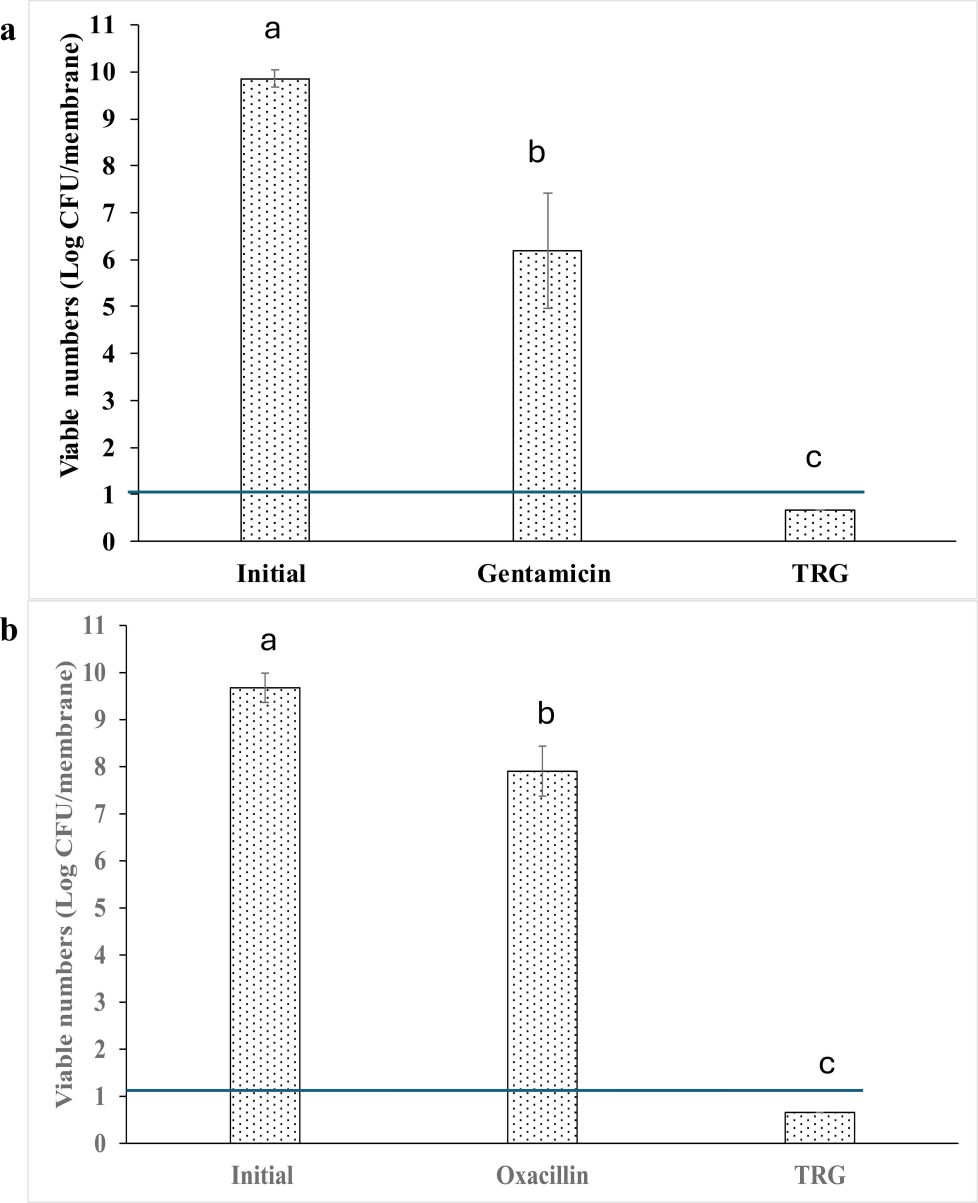
Effect of TRG on antibiotic tolerant biofilm of **(a)***P. aeruginosa* and **(b)***S. aureus* using colony biofilm model. Horizontal line indicates detection limit. Data analysis was performed using one-way ANOVA followed by Tukey’s post-hoc test. Mean values marked with different letters indicate statistically significant differences.

The MIC of gentamicin against *P. aeruginosa* was 6.25 µg/mL, while the MIC of oxacillin against *S. aureus* was 0.063 µg/mL. After 72 hours, the viable numbers of *P. aeruginosa* and *S. aureus* biofilms were ≥ 9.5 log CFU/membrane. Following antibiotic treatment at 50× MIC, viable counts remained at 6.2 log CFU/membrane for *P. aeruginosa* and 7.9 log CFU/membrane for *S. aureus*, indicating a high level of tolerance. (**Figure 1**). TRG treatment reduced the viable numbers of both organisms to below the detection limit at 24h.

### 3.2 Effect on total biofilm using porcine skin explant model

**Figure 2:**
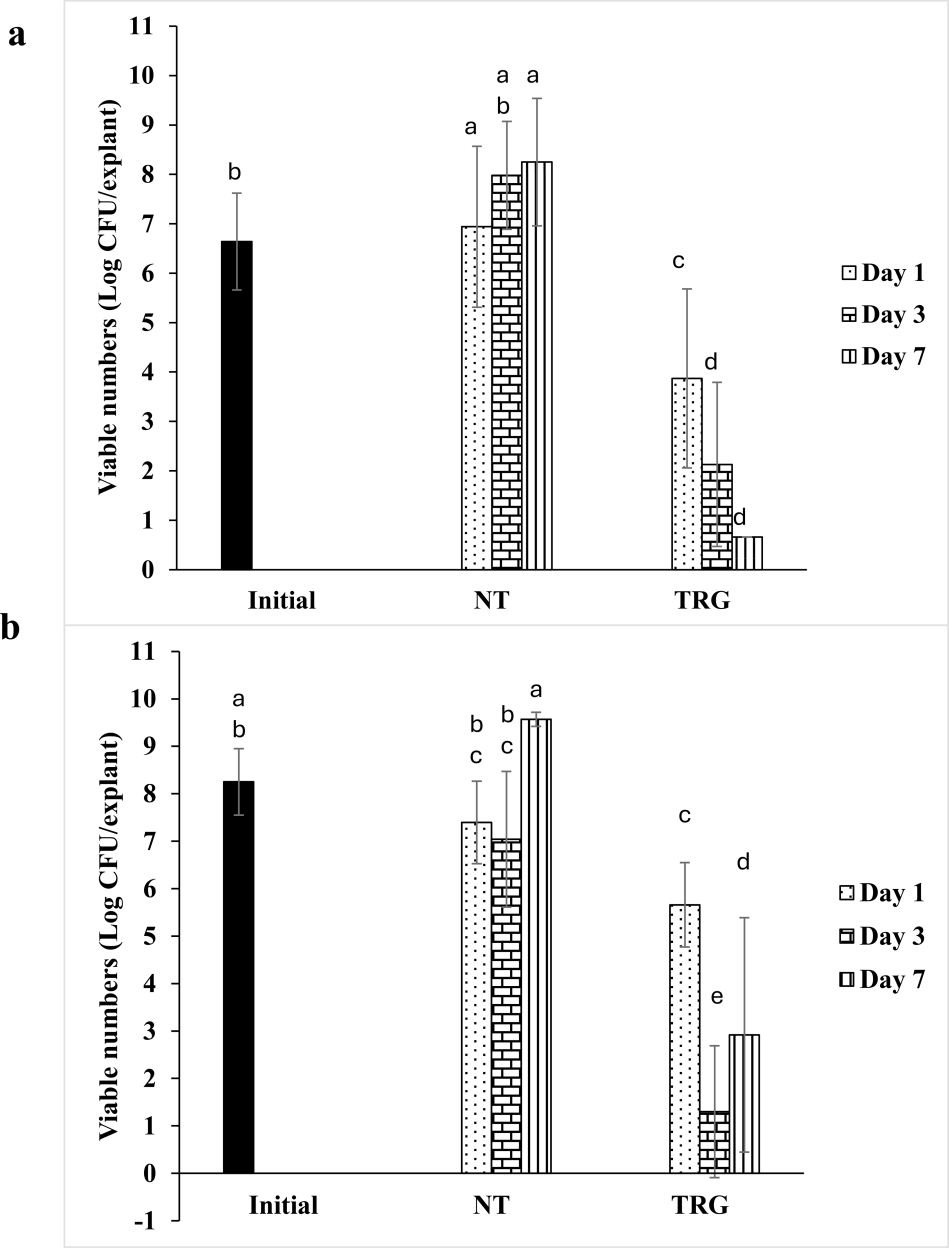
Effect of TRG on total biofilm of **(a)***P. aeruginosa* and **(b)***S. aureus* using porcine skin explant biofilm. Data analysis was performed using one-way ANOVA followed by Tukey’s post-hoc test. Mean values marked with different letters indicate statistically significant differences.

After 72 hours, the viable numbers of *P. aeruginosa* biofilm were 6.6 log CFU (**Figure 2A**), while the numbers in the non-treated control were 6.9, 8.0 and 8.3 log CFU on day 1, 3 and 7, respectively. A single TRG treatment reduced viable numbers by 3.1, 5.9, and 7.6 log CFU compared to corresponding non-treated control on days 1, 3, and 7, respectively. For *S. aureus*, the initial and non-treated control biofilm viable numbers ranged between 7 and 9.5 log CFU; however, these differences were not statistically significant (**Figure 2B**). A single TRG treatment resulted in reductions of 1.7, 5.7, and 6.7 log CFU compared to corresponding non-treated control on days 1, 3, and 7, respectively.

### 3.3 Effect on antibiotic tolerant biofilm using porcine skin explant model

**Figure 3:**
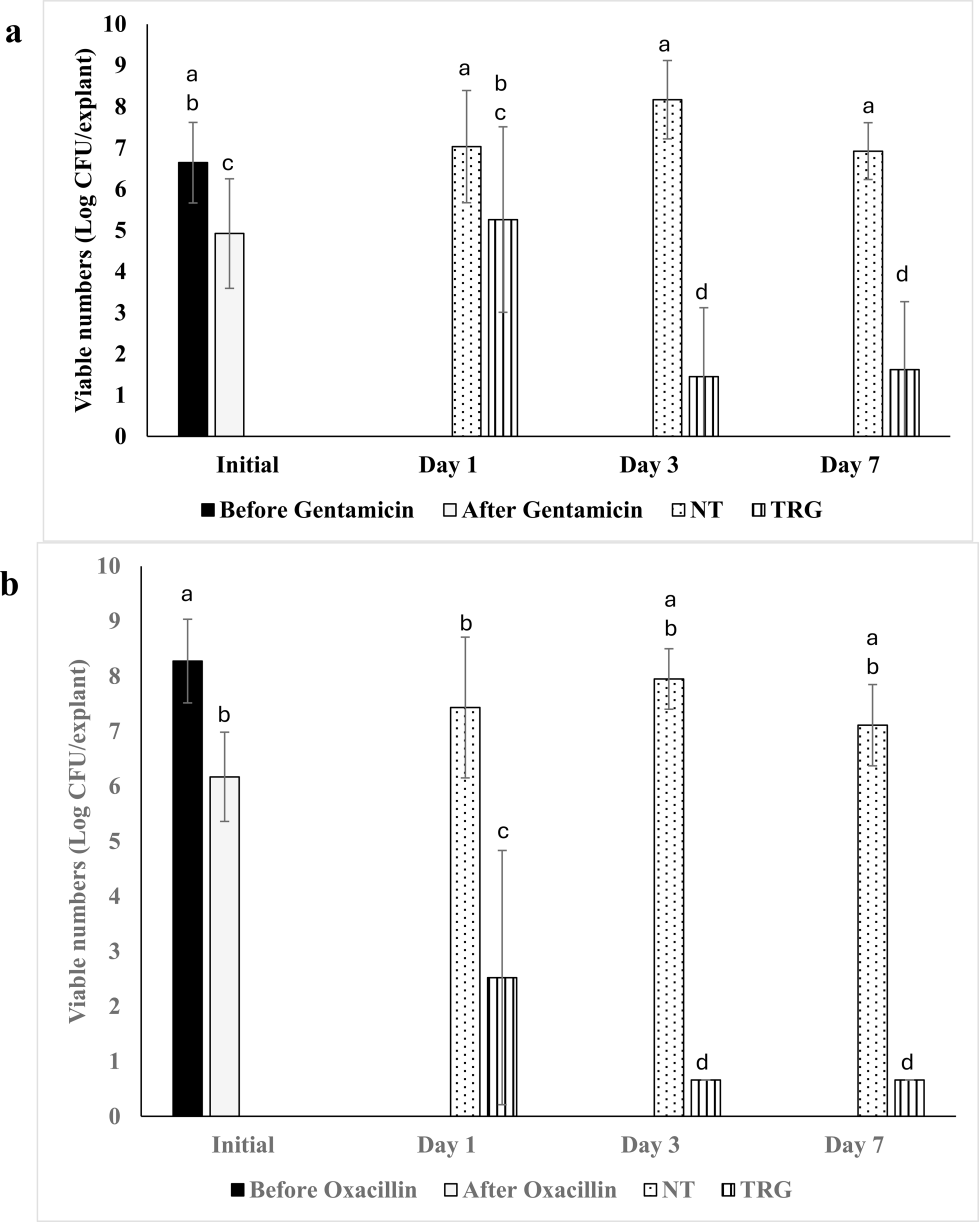
Effect of TRG on antibiotic tolerant biofilm of **(a)***P. aeruginosa* and **(b)***S. aureus* using porcine skin explant biofilm. Data analysis was performed using one-way ANOVA followed by Tukey’s post-hoc test. Mean values marked with different letters indicate statistically significant differences.

Antibiotic treatment of mature (72h) biofilms resulted in approximately a 2 log CFU reduction in both *P. aeruginosa* and *S. aureus*, leading to the survival of 5 and 6 log CFU, respectively (**Figure 3**). A single TRG application caused a 3.5 and 5.5 log CFU reduction in antibiotic-tolerant biofilm cells of *P. aeruginosa* and *S. aureus*, respectively, by day 3, with no further changes in viable numbers observed by day 7. Meanwhile, viable numbers of both organisms increased by 1–2 log CFU in the non-treated controls.

## 4 Discussions

This study evaluated the effect of TRG (revyve^®^) on antibiotic-tolerant biofilms of *P. aeruginosa* and *S. aureus* using colony and porcine skin explant models. The colony biofilm method, which has previously been used to assess wound care products (Hammond et al., 2011; Qu et al., 2020) mimics the environmental conditions necessary for biofilm formation on wound surfaces. In this model, microorganisms grow on a filter membrane supplied with nutrients diffusing from below and are exposed to oxygen without shear stress. The porcine skin explant model is considered highly representative of human skin, providing host proteins and extracellular matrix components that are not typically present in standard *in vitro* models. In addition, it provides a three-dimensional structure for biofilm growth, simulating the wound environment where biofilms often develop across different tissue layers (Peterson and Westgate, 2022; Sullivan et al., 2001; Yang et al., 2013).

The porcine skin explant model was initially developed using *S. aureus* and *P. aeruginosa* with known resistance markers (Yang et al., 2013). In contrast, the strains used in this study do not possess any known resistance markers (ATCC, 2024, 2025). This allows for the assessment of biofilm-mediated antibiotic tolerance in two different models without the confounding influence of genetically mediated antibiotic resistance. The oxacillin MIC for *S. aureus* was below the susceptible breakpoint (CDER, 2022), whereas the gentamicin MIC for *P. aeruginosa* fell between the susceptible and intermediate-resistance breakpoints (Humphries, 2023).

Generally, it has been reported that biofilms are up to 1000 times more tolerant to antimicrobial agents than their planktonic counterparts (Shree et al., 2023). *S. aureus* and *P. aeruginosa* biofilms developed using a wound biofilm model have been shown to tolerate 50-time higher concentration of bleach than planktonic culture (Sun et al., 2008). Other studies have utilized 5-50x antibiotic MIC to isolate persister cell populations (Peyrusson et al., 2020; Pereira et al., 2025; Lin et al., 2022; Khandekar et al., 2018). The possibility that surviving cells after antibiotic exposure were resistant mutants rather than antibiotic-tolerant persister cells is highly unlikely based on established mutation frequencies. Spontaneous resistance mutations typically occur at rates of 10^−7^–10^−10^ in bacteria (Martinez and Baquero, 2000) and reported frequencies for *P. aeruginosa* range from 7.5×10^−7^ to 1.1×10^−8^ (Yasuda et al., 2023). Using these values, the estimated number of resistant mutants present before antibiotic treatment would be only 0.048–3.27 cells in *P. aeruginosa* and 1.89–18.87 cells in *S. aureus*, based on the mean biofilm populations at 72h. These estimates were several orders of magnitude lower than the post-treatment survivor counts (≥ 5 log CFU and ≥ 6 log CFU, respectively in both models). Thus, the surviving populations cannot be explained by spontaneous mutation. Instead, their magnitude strongly supports the presence of antibiotic-tolerant persister cells formed during biofilm maturation.

In a previous study, the effect of TRG on total biofilms formed by *S. aureus*, *P. aeruginosa*, methicillin-resistant *S. aureus* (MRSA) and other wound-associated pathogens was evaluated using colony biofilm model (Visvalingam et al., 2026). TRG was found to reduce the viable counts of *S. aureus*, MRSA and other pathogens by ≥ 6 log CFU within 72 hours, while *P. aeruginosa* counts decreased by approximately 4 log CFU at 24h with no further reduction thereafter. In contrast, 50x antibiotic tolerant sub-population of *S. aureus* and *P. aeruginosa* biofilms were effectively inactivated by TRG in 24h to below detection limit, corresponding to a ≥ 6 log CFU reduction.

Since the antibiotic concentrations used to grow porcine skin explant biofilms of *P. aeruginosa* and *S. aureus* were 10× and 25× the MIC of gentamicin and oxacillin, respectively, the same concentrations were used to select antibiotic-tolerant biofilms. It should be noted that the addition of appropriate antibiotic concentration to the agar was necessary in the porcine skin explant model to prevent biofilm spread from the tissue surface onto the surrounding agar (Yang et al., 2013). In contrast, this was not required in the colony biofilm model, where the growth surface is a nitrocellulose membrane with a pore size of 0.2 µm. This membrane structure inherently prevents bacterial penetration and migration onto the agar surface, eliminating the need for antibiotic supplementation in the underlying medium.

In the porcine skin explant model, viable counts of *P. aeruginosa* total biofilm progressively declined to below the detection limit by day 7 of TRG treatment, while *S. aureus* counts were reduced by ≥ 5 log CFU by day 3, with no significant changes thereafter. In contrast, the antibiotic-tolerant cells within the biofilms of both organisms showed a stable reduction approaching the detection limit by day 3 of TRG treatment. Due to the substantial differences between the two models used in this study, including growth surface, architecture, and overall physical structure, statistical comparison of their outcomes would be inappropriate. However, observation from both models showed that the number of viable cells in the total biofilm and the number of antibiotic-tolerant cells in the biofilm varied between the two models. This is understandable, as variability in the growth surface, substrate, and oxygen gradient in each model can affect metabolic activity, which in turn influences the proportion of slow-growing and dormant persister cells (Uruén et al., 2020; Shree et al., 2023; Williams et al., 2019). Furthermore, the time required to inactivate antibiotic-tolerant biofilm cells was slightly longer for the porcine skin model than for the colony model (72h vs. 24h). This was possibly due to the three-dimensional nature of the porcine skin explant, which allows biofilms to grow at different depths within the explant, resulting in slower penetration of the gel components (Yang et al., 2013). Despite these variations, the results clearly demonstrate that TRG was effective in inactivating antibiotic-tolerant biofilms.

TRG is formulated with poloxamer 407, citrate, ethylenediaminetetraacetic acid (EDTA), and polyhexanide (PHMB). Unlike most antibiotics that typically act on a single cellular target (Uruén et al., 2020; Shree et al., 2023), PHMB has multi-mode of antimicrobial action. As a cationic antimicrobial, PHMB interacts with negatively charged components of bacterial membranes, increasing membrane permeability and compromising membrane integrity. Once inside the microbial cell, PHMB interferes with metabolic processes and binds to DNA, leading to cell inactivation (Hübner and Kramer, 2010). As a result, the likelihood of resistance development is minimal, further supported by over six decades of use without any reports of resistant strains (Gray et al., 2010). Poloxamer 407, a hydrophilic, non-ionic surfactant, has been shown to offer several advantages for wound healing such as facilitating wound cleansing and disrupting biofilms and promoting cell repair and viability. Additionally, its thermo-gelling nature enables the sustained release of antimicrobial agents (Percival et al., 2018, 2019; Ricci et al., 2005; An et al., 2021). The antimicrobial activity and pH of wound dressings is further modulated by incorporating citric acid and ethylenediaminetetraacetic acid (EDTA), metal ion chelators known to enhance antimicrobial and antibiofilm property. Both compounds have demonstrated positive effects on wound healing outcomes (Jensen et al., 1998; Finnegan and Percival, 2015; Nagoba et al., 2011; Malu et al., 2014). In conjunction with the evidence presented above and as evidenced by the efficacy of TRG against both total biofilms and antibiotic-tolerant biofilms in two wound biofilm models, the components of TRG collectively disrupt biofilm structure, enabling the antimicrobial agents to inactivate pathogens and maintain antibiofilm activity for up to seven days, thereby preventing biofilm regrowth. A recent study with TRG demonstrated that the formulation is biocompatible, does not induce cytotoxic effects, and provides sustained antimicrobial and antibiofilm activity (Visvalingam et al., 2026). These findings provide strong support for the potential use of TRG as a valuable tool in the management of biofilms in chronic wounds. Furthermore, dressing changes are typically performed at 3–7-day intervals (He et al., 2026; Britto et al., 2025). The significant reduction in both total biofilm and antibiotic-tolerant biofilm achieved by TRG by day 3, along with the maintenance of reduced viable counts for up to 7 days, could provide clinicians with increased flexibility in dressing-change intervals and can be readily integrated into routine wound-care regimens. However, further validation through *in vivo* or clinical studies is warranted.

In conclusion, this study provides evidence that TRG is effective in inactivating mature biofilms containing a large proportion of antibiotic-tolerant cells and may serve as a valuable tool for controlling biofilms in chronic and burn wounds.

## Data Availability

The original contributions presented in the study are included in the article/supplementary material. Further inquiries can be directed to the corresponding author.
